# Life situation and psychosocial care of adolescent and young adult (AYA) cancer patients – study protocol of a 12-month prospective longitudinal study

**DOI:** 10.1186/s12885-017-3077-z

**Published:** 2017-01-28

**Authors:** Katja Leuteritz, Michael Friedrich, Erik Nowe, Annekathrin Sender, Yve Stöbel-Richter, Kristina Geue

**Affiliations:** 1 Department of Mental Health, Medical Psychology & Medical Sociology, University of Leipzig, Philipp-Rosenthal-Str. 55, Leipzig, 04103 Germany; 2 University of Zittau / Goerlitz, Faculty of Management and Cultural Sciences, P. O. Box 30 06 48, Goerlitz, 02811 Germany

**Keywords:** Adolescent and young adult, AYA, Cancer, Longitudinal study, Psychological distress, Quality of life

## Abstract

**Background:**

In recent years, there has been an increased research focus on adolescent and young adult (AYA) cancer patients. Few longitudinal studies have taken into consideration the specifics of their life situation and the status of psychosocial care services for this population. Our ongoing study aims to determine the psychosocial life and supportive care situation of AYA cancer patients, to describe risk groups, and to develop recommendations for their psycho-oncological care and support.

**Methods:**

The AYA-Leipzig study (AYA-LE) is a German prospective, longitudinal, study examining AYAs´ life situation (e.g. psychological distress, quality of life) and psychosocial care (e.g. evaluation and preferences, support needs) using two measurement points, namely, upon acute treatment completion (baseline) and 12 months later. *N* = 577 AYA cancer patients aged between 18 and 39 years at diagnosis, and representing all major tumor entities fill out a standardized questionnaire (online or by post), mainly based on validated instruments. AYA-specific concerns (e.g. family planning, sexual and reproductive health, social support, health behavior) will explicitly be considered. Participants are recruited in 16 German acute care hospitals, four rehabilitation clinics, and from two German state tumor registries.

**Discussion:**

In summary, our longitudinal study will create a large database encompassing all malignant tumor entities and including detailed information about the distress and quality of life, specific problems, and specific support needs of AYA cancer patients at two different points in time post-diagnosis. The information we gather about existing psychosocial care and patient preferences and desires concerning psycho-oncological care will be used to develop recommendations for psycho-oncological care providers.

## Background

Over the last decades, the incidence of cancer in “Adolescents and Young Adults” (AYAs) has increased both in Europe and North America [1–3]. The National Cancer Institute defined the age range of AYA as being 15 to 39 years at the time of diagnosis [4]. Each year about 15.000 young people in Germany are diagnosed with cancer [5]. AYA patients differ in terms of biological, epidemiological, and clinical characteristics from other patient groups both younger and older than them [6]. With a 10-year overall survival rate of around 80%, 20- to 39-year olds’ chances of recovery are well above average [7].

While there are a number of studies on the psychosocial life of cancer patients who are either children or further into adulthood, so far, little empirical data has been collected on patients between the ages of 15 and 39 years old [8–10]. The few studies that do exist show that AYA cancer patients experience many specific sequelae after cancer diagnosis and treatment such as: loss of fertility [11, 12], hair loss, other changes in body image, and fatique [13, 14], as well as further difficulties related to social relationships, employment, educational attainment, and financial stability [15, 16]. Regarding mental health, the majority of the existing studies have shown that adolescent cancer patients have increased anxiety, depression, and distress compared to their healthy peers and the general public [10, 17, 18]. Those concerns often result in impaired quality of life (QoL). In a recent systematic review by Quinn et al. [19] about QoL in AYA cancer patients, nearly 35 studies (29 of which were mainly done in the USA and Canada) concluded, that AYA cancer survivors are more likely to have poorer QoL compared with the age-matched general population and older cancer survivors. In this review only 11 out of 35 studies included all cancer types, and only three of those 11 had satisfactory sample sizes (greater than 100) [11, 14, 20]. Consequently, Warner et al. [16] have highlighted the need for AYA-studies with more heterogeneous cancer samples that would allow for comparing subgroups.

Previous studies have found that AYA cancer patients have different psychosocial and medical needs during acute treatment than they do in subsequent phases of rehabilitation or after-care [17, 21]. Consequently, it is essential that well designed, prospective, controlled studies of QoL among AYAs be conducted to examine outcomes across the survivorship continuum in specific cancer types [8]. To our knowledge, only three longitudinal studies have been done so far to examine AYA cancer patients’ psychosocial situation [22–24].

Furthermore, in their recent report about the current state of science associated with cancer among AYAs, Smith et al. [8] as well as Nightingale et al. [25] state that the instruments that have been used, to date, neglect some important and unique concerns of AYA cancer survivors. Qualitative research has found the themes health behavior, reproductive and sexual health, and social support by family and friends to be issues that have a profound impact on AYA cancer patients’ QoL [26].

Our study aims to examine a large sample of AYA cancer patients diagnosed with a cross-section of malignant cancer types by focusing on their psychological distress, QoL, psychosocial living situation, and use of psychosocial support services. Within a longitudinal design, we will also consider AYA-specific concerns like health behavior, family planning and sexual and reproductive health.

### Research questions

Following are the research questions of the AYA-Leipzig study (AYA-LE) focusing on AYA cancer patients across all major tumor entities both upon completion of acute treatment and one year later within the in- and outpatient oncological health care and rehabilitation settings in Germany:

- What is the frequency of psychological distress on young adults with cancer?
- How is the degree of quality of life in young adults with cancer?
- How is the global life satisfaction and life satisfaction in specific life domains in young adults with cancer?

Secondary research questions are:

- What medical and psycho-social characteristics (e.g. gender, diagnosis group, age group, medical treatment group) are associated with distress and quality of life in young adults with cancer?
- How is the psychosocial supportive care situation (utilization of supportive care services, unmet supportive care needs, psychosocial support) in young adults with cancer?
- How do psychological distress, quality of life and the psychosocial supportive care situation change over time (upon completion of acute treatment to one year later)?

Recommendations for the AYA psycho-oncological care shall be developed on the basis of the results.

## Methods

### Study design

**Fig. 1:**
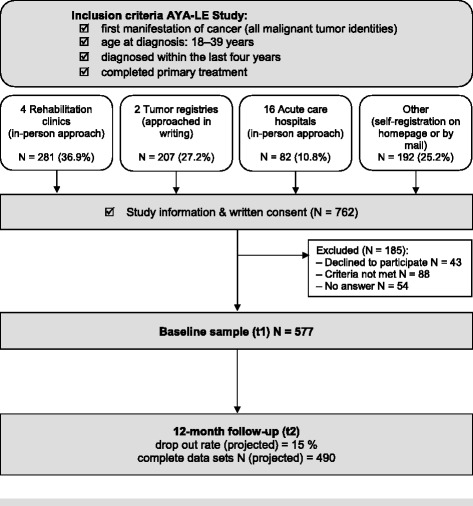
Study design of the AYA-LE study

The AYA-LE study has been implemented as a prospective longitudinal epidemiological study with two measuring points. From May 2014 to December 2015 cancer patients between 18 and 39 years old at diagnosis completed the baseline survey upon completion of their acute treatment. The 12-month follow up measurements are currently ongoing and will be completed at the end of December 2016. The study is funded by the German Cancer Aid and will be completed on 31 March 2017 (Fig. 1).

### Sample size calculation

In the projected recruitment period of 18 months the AYA-LE Study has been initiating cooperation agreements with four rehabilitations clinics, two tumor registries and 16 acute care hospitals. The four rehabilitation clinics treat in sum *n* = 400 eligible patients 18-39 years of age. In the light of having the possibility to contact patients more than one time during rehabilitation by a stable contact person within the team of the rehabilitation clinic it is expected that at least 80% of those patients (n_rehabilitation clinics_ =320) will complete the questionnaire. The number of eligible patients treated in the acute care hospitals differs quite widely from one another between *n* = 4 to *n* = 15 patients. That is why our estimation of the eligible total number in the 16 acute care hospitals is *n* = 150. In the face of the fact that recruitment in acute hospitals is a greater challenge because of the relatively short retention time in acute hospitals it is expected that at least 50% of those patients (n_acute care hospitals_ = 75) will complete the questionnaire. Projected data from the two tumor registries is *n* = 650 eligible participants. It is expected that at least 50% (n_tumor registries_ = 325) of those will complete the questionnaire. Further, there will be the possibility for self-registration for the study estimated with approximately n_self-registration_ = 50 participants. In view of experiences of past studies corresponding failure rates (e.g. patients contacted several times in different clinics) must be taken into account with 10%. Thus, the total expected baseline sample size is n_baseline_ = 693.

With a projected drop-out/failure rate between baseline and follow-up of 20%, we expect around n_12-month follow-up_ = 554 complete data sets at the 12-month follow-up.

## Participants

### Inclusion criteria

Participants must meet the following inclusion criteria to attend in the study:

1. age at diagnosis: 18 – 39 years. (Our study aims to draw conclusions about patients treated in adult oncology departments. In Germany, patients younger than 18 years are treated in pediatric oncology units, and patients over 18 years are treated in adult oncology units. That is why we focused on 18 to 39 years olds.);
2. first manifestation of cancer (all malignant tumor identities C00-C97 without C44);
3. diagnosed within the last 4 years; and
4. completed acute medical treatment.

### Exclusion criteria

AYAs are excluded from study participation if an initial screening of their data reveals that:

- they are unable to speak German;
- they are physically or cognitively not able to participate in the survey; or
- they did not provide written consent.

### Patient recruitment and data collection

The study received research ethics committee approval (Ref.-Nr. 372-13-16122013) from the responsible institutional ethics board of the medical faculty of the University of Leipzig. Principles of good research practice are strictly adhered to in this study.

The recruitment of participants is carried out throughout Germany in cooperation with 16 oncological acute care hospitals, four (cancer) rehabilitation clinics, and two tumor registries. Candidates who meet the inclusion criteria are informed about the study (carried out by trained staff) and are invited to participate. Interested participants are given information about the research project via flyers, posters, the project’s website, and a facebook page with a direct link for applying for the study. After consenting to take part in the study (patient master sheet and consent form), participants are provided either with a link for answering the standardized study questionnaire online with Lime Survey [27] or, if so desired, a hard copy of the questionnaire sent by post. To maximize the participation rate, we use different strategies: First, participants receive a compensation fee of 10 Euros for completing the questionnaire at each measuring point. After initially inviting participants, we remind them about the survey by mail or telephone at two-week intervals, but no more than three times total. Additionally, there is a broad panel management; all of the participants are receiving Christmas and Easter greetings and we regularly post AYA-specific information on the study’s Facebook page. Regarding the follow-up measurement, participants are to be contacted 11 months after baseline and to be invited to again complete either an online or a hard copy version of the questionnaire. Reminders are given the same way they are at baseline. The follow-up survey is currently underway.

### Study measures

Table 1 gives an overview of the assessment schedule for the two time points. We pre-tested both the online and hard copy questionnaire formats for length and clarity with AYA before rolling out the study.

**Table 1:** Assessment schedule for the AYA-LE study for both survey time points

Topic	Measurement instruments at baseline and follow-up
*Life situation*	
Psychological distress	Hospital Anxiety and Depression Scale (HADS) NCCN Distress thermometer
Quality of life (QoL)	Questions on life satisfaction (FLZ-M) The European Organization for Research and Treatment of Cancer Quality of Life Questionnaire-Core 30 (EORTC QLQ-C30)
Fatique	The European Organization for Research and Treatment of Cancer Quality of Life Questionnaire- Cancer Related Fatique (EORTC QLQ-FA12)
Sexuality	Questionnaire on Life Satisfaction (FLZ, scale *partnership/sexuality)*
Employment and work ability	self-developed
Health behavior	Items based on Questionnaire of Multiple Health Behavior (MHB-39)
Self-regulation	Global-Motivation Scale (GMS-18D) Perceived Adjustment to Chronic Illness Scale (PACIS)
*Psychosocial care*	
Supportive care needs	Supportive Care Needs Survey Questionnaire (SCNS-SF34-G)
Psychosocial support	Illness-specific Social Support Scale Short Version-8 (ISSS-8)
Preferences and evaluation of psychosocial care	self-developed
*Other*	
Socio-demographic variables	date of birth, gender, nationality, marital status, partnership, having children, housing situation, net monthly household income, highest educational degree, state of employment, religion
Medical variables	diagnosis, metastasis, recurrence, type of medical treatment, co-morbid diseases, participation in vocational rehabilitation program

The instruments used are described below:

#### Hospital Anxiety and Depression Scale - German version (HADS)

The HADS is a validated screening instrument for anxiety and depression in somatically ill patients [28] and excludes symptoms that may arise from somatic aspects of illness. The instrument consists of 14 items rated on a 4-point Likert scale ranging from 0 to 3 comprising an anxiety and depression subscale. Individual scores can be calculated for each subscale (ranging from 0–21) as well as for a total score [29]. There are cutoffs for the subscales and the total score. The internal consistency was appropriate in most cases with Cronbachs alpha values above 0.80 [30].

### NCCN distress thermometer

The Distress Thermometer is a valid and reliable measurement instrument for screening psychological distress in patients with cancer. Initially developed by the National Comprehensive Cancer Network (NCCN) [31], the German version was adapted by Mehnert et al. [32]. The measure contains a single-item visual analogue scale ranging from 0 (“no distress”) to 10 (“extreme distress”) to quantify the global level of distress along with a standardized symptom checklist. A score of 5 or higher on the visual analogue scale is recommended as a cutoff for indicating clinically significant levels of distress [32].

#### Questions on Life Satisfaction (FLZ-M)

The instrument Questions on Life Satisfaction (FLZ-M) [33] evaluates the responder´s subjective quality of life. The questionnaire consists of different modules: The module "general life satisfaction" has eight items based on the following areas of life: *friends & acquaintances, leisure activities & hobbies, health, income & financial security, work & profession, housing situation, family & children, and partnership & sexuality.* We measured *partnership & sexuality* using two separate items. This is also how we processed the scale *family & children.* Participants’ “subjective satisfaction” with the resulting 10 areas of life are to be rated on a 5-point Likert-Scale from 1 (“unsatisfied“) to 5 (“very satisfied“). A global satisfaction score can be tallied. The FLZ-M is a valid instrument which favors the subjective valuation of functionality in different areas of life. Furthermore, we assessed post-cancer diagnosis changes in the ten aforementioned life domains, using a five point Likert Scale (ranging from 1 (“not at all”) to 5 (“very much”)).

#### European Organization for the Research and Treatment of Cancer Quality of Life Questionnaire-Core 30(EORTC QLQ-C30)

The EORTC QLQ-C30 is used to measure patients’ quality of life on five function scales (physical, role, emotional, cognitive, and social), three symptom scales (fatigue, nausea, pain), six symptom scales (e.g. dyspnea, insomnia, financial difficulties), and a two-item global health status/QoL scale [34]. The EORTC QLQ–C30 consists of 30 items scored on 4-point Likert scales, ranging from 1 (“not at all”) to 4 (“very much”). All scales are scored from 0 to 100. Acceptable values for a high reliability (α > .70) and a good construct validity have been demonstrated for all scales of the German version [35].

#### European Organization for the Research and Treatment of Cancer Quality of Life Questionnaire- Cancer elated Fatique (EORTC QLQ-FA12)

The FA12 is a module of 12 items that can be used in addition to the quality of life questionnaire EORTC QLQ-C30. In accordance with the EORTC QLQ-C30, it is scored using 4-point Likert scales. The FA12 scores are then converted to a 0–100 range. The original publication of the instrument [36, 37] describes, along with the final phase III module EORTC QLQ-FA12, several properties of a preceding version with 15 items.

#### The Life Satisfaction Questionnaire (FLZ) – sexuality scale

The FLZ is a German instrument for evaluating life satisfaction in ten life domains. Every subscale is comprised of seven items rated using a seven-point-Likert-scale ranging from 1 (“very unsatisfied*”)* to 7 (“very satisfied*”*) that are summarized into a total scale score (7 to 49). Higher scores indicate higher levels of satisfaction with physical attraction, sexual efficiency, sexual contacts, and sexual reactions. The internal consistency for the sexuality scale was α = 0.92 [38].

#### Employment factors and work ability

Occupational and work-related characteristics are assessed using scales developed and psychometrically evaluated by Bürger et al. [39]. Occupational information includes (1) professional status, (2) work ability and periods of sick leave absence, (3) grade of responsibility, (4) hours worked per week, (5) time limited work contract (6), perceived threat of job loss, (7) self-perceived probability of working again. At follow-up, we also ask about physical demands at work and workplace adjustments. Self-perceived total work ability is rated [40]. All those questions have different answer formats.

#### Multiple health behavior (MHB-39)

Selected items of our survey are based on the questionnaire Multiple Health Behavior (MHB-39 [41]), an instrument designed for assessing habitual health-related behavior. We chose 15 items based on their content and statistical relevance to our study. The items are to be rated on a 5-point Likert-type scale from 1 (“never”) to 5 (“ever”) and assigned to six different categories (active lifestyle, medical compliance, substance avoidance, personal safety-related behavior, nutrition, and hygiene).

#### Global Motivation Scale (GMS)

The 18-item Global Motivation Scale (GMS) assesses six different types of motivation: (1) intrinsic motivation; extrinsic motivation: (2) integrated, (3) identified, (4) introjected and (5) external regulation) and (6) amotivation as described by Self-Determination-Theory [42]. The subscale for intrinsic motivation consists of three items distinguishing between intrinsic motivation toward knowledge, stimulation and accomplishment. The Global Motivation Scale was translated from English to German. Every subscale consists of three items rated on a 7-point Likert-type scale from 1 (“not agree at all”) to 7 (“completely agree”) and can be computed as a sum score or as a mean of the three items. Cronbachs α of the subscales ranges from 0.69 to 0.82 [43].

#### Perceived Adjustment to Chronic Illness Scale (PACIS)

Perceived adjustment/coping ("How much effort does it cost you to cope with your illness?" [“none*”* to *“*a great deal*”*]) will be assessed with a single-item linear analogue self-assessment scale (1 to 100) [44], previously validated in several cancer populations.

#### Supportive Care Needs Survey Questionnaire (SCNS-SF34-G)

The SCNS-SF34 is a well-established self-report questionnaire for measuring type and amount of perceived needs among oncological patients in five dimensions: (1) health system and information, (2) psychological state, (3) physical and daily living, (4) patient care and support, and (5) sexuality. Participants indicate their level of need for help with each item over the past month, measured on a five-point scale from 1 (“no need/not applicable”) to 5 (“high need”). Subscale scores on the five dimensions are to be calculated by summing individual item scores and rescaling to a 0 – 100 range, with higher scores indicating a greater extent of need. We added two supplementary AYA-relevant items to the scale, having to do with fertility and the desire to have children. Cronbachs alpha values ranged from 0.82 to 0.94 [45].

#### Illness-Specific Social Support Scale short version-8 (ISSS-8)

The Illness-Specific Social Support Scale (ISSS) was originally developed by Revenson and Schiaffino [46], and has been adapted to the German language by Ramm and Hasenbring [47]. The newly developed 8-item version [48] is comprised of two scales: “positive support” (4 items) and “detrimental interactions” (4 items). Items are scored on a 5-point Likert scale ranging from 0 (“never”) to 4 (“always”). The two scales show internal consistencies with Cronbachs alpha = 0.88 and 0.68.

#### Preferences and evaluation of psychosocial care

We measure underused mental health care supply services and AYAs’ actual mental health care needs with three self-developed items. In addition to that, we use a self-designed questionnaire to measure patients’ use and satisfaction with mental health care and to find out what they want in these areas both during acute disease treatment and afterwards. Patients are asked to distinguish between inpatient and outpatient care when responding. Initially, participants indicate which psychosocial care services (e.g. social and legal advice, psychological counseling, and/or self-help groups) they have taken advantage of over the last 12 months and how often. They are also asked to use a five-point Likert scale to specify how useful the service was, and in the event that they did not use the service, why this was the case. Participants also use a five-point Likert scale to rate the importance of and their satisfaction with services addressing specific topics (e.g. anxiety, effects of the disease on family, coping strategies, sexuality, and fertility).

### Data analyses

We use the Statistical Package for the Social Sciences 23 (SPSS, by IBM) for all quantitative statistical data analyses.

The data collected in this study will be described in terms of mean, standard deviation, median, minimum and maximum. Frequencies of the primary outcomes psychological distress and QoL will be reported in combination with confidence intervals. Histograms and boxplots will be used for graphical representation. For comparison of two groups (e.g. women vs. men; diagnosis group 1 vs. diagnosis group 2) with respect to a categorial target variable, cross tables and Fisher tests are calculated. T-tests (with normal distribution) or Mann-Whitney U- tests (in case of absence of a normal distribution) will be used for the comparison of two groups with respect to a continuous target variable.

Exploration of associated variables and group comparisons (e.g. men vs. women, age groups, group of cancer diagnosis, other relevant variables), will be performed using analysis of variance (ANOVA), multiple linear regression (for metric dependent variables) and multiple logistic regression analysis (for binary dependent variables). All models will be adjusted for relevant confounders. We aim to calculate models including approximately five binary risk-factors for both measurement time points (baseline and follow up) focusing on different psycho-oncological characteristics (e.g. psychological distress, quality of life).

To ensure sufficient power we will calculate a post-hoc power analysis with G*Power 3.1.7 for an ANOVA with repeated measures and effects between factors, because tests of within-effects and effects of within-between-interactions have more power anyway. For two measurements and 10 groups we will use a type-I-error probability of α = 0.05, an effect size of *f* = 0.02 (this corresponds to a Cohen’s d of 0.4 and is somewhat smaller than a medium effect of d = 0.5) and the correlation between measurements is assumed to be strong with *r* = 0.5. For a sample size of *n* = 500 we have a Power of 1-β = 0.97 and for *n* = 400 the power would be 1-β = 0.92. For a sufficiently powered analysis we would need at least a power of 1-β = 0.80, so there is some room for slight changes in parameters that would result in lower power (e.g. higher correlations between measurements, more binary variables or smaller effect sizes).

## Results

### Sample

After being screened for inclusion criteria, *n* = 762 participants gave written consent to participate. Of these candidates, *n* = 185 could not be included either because they later declined to participate (*n* = 43), did not meet inclusion criteria in a secondary verification screening (*n* = 88), or did not complete the questionnaire (*n* = 54). Thus, *n* = 577 participants form the baseline sample (t1). The 12-month follow-up is currently being conducted. Figure 1 (shown above) shows the study design and the enrollment of the study participants.

### Baseline characteristics of the sample (t1)

Most of the participants are female (*n* = 425, 73.7%). The mean age at diagnosis is 29.3 years (SD = 6.09). Most participants are in partnership (68.1%). A third of the participants (*n* = 195, 33.8%) has one or more children. Most of the participants completed the hard copy of the questionnaire at baseline (*n* = 365, 61.7%).

The most common diagnoses among the included participants is breast cancer (*n* = 150, 26.0%) and Hodgkin´s lymphoma (*n* = 99, 17.2%). The rarest entity is melanoma (*n* = 19, 3.3%). The average length of time passed since diagnosis is 11.9 months (Table 2).

**Table 2:** Baseline characteristics (t1) of the AYA-LE sample

Total *n* = 577	Valid	
	n	(%)
*Gender*: female	425 29.3 391	(73.7) (6.09) (68.1)
*Age at diagnosis* Mean (SD); Min-Max:		
*Partnership*: (yes)		
*Highest educational degree*:		
No educational degree Basic educational degree (<10 years) Secondary educational degree (10 years) Highschool degree (>10 years)	6 37 190 340	(1.0) (6.5) (33.2) (59.3)
*Children*: (yes)	195	(33.8)
*Cancer diagnosis*		
[C50] Breast Cancer [C51-C57] Gynecological Cancer [C62] Testicular Cancer [C73] Thyroid Cancer [C43] Melanoma [C81] Hodgkin´s Lymphoma [C82-C90] Non-Hodgkin´s Lymphoma [C91-C95] Haematological Cancer [C40-C41, C46-C49] Sarcoma [C15-C26] Gastrointestinal cancer [other C] Others	150 51 50 32 19 99 42 40 26 29 39	(26.0) (8.8) (8.7) (5.5) (3.3) (17.2) (7.3) (6.9) (4.5) (5.0) (6.8)
*Time since diagnosis in months* Mean (SD); Min-Max:	11.9 (7.99); 1.0 - 44.4	
*Medical therapies* *(Multiple answers possible):*		
Surgery Radiation Chemotherapy Chemoradiotherapy	427 250 228 215	(74.0) (43.3) (39.5) (37.3)
*Sick leave at baseline*: (yes)	321	(57.0)

## Discussion

The AYA-LE Study aims to examine a large sample of AYA young adult cancer patients diagnosed with a cross-section of malignant cancer types by focusing on their psychological distress, QoL and psychosocial care using a longitudinal study design. The study will shed light on what factors determine psychological distress, quality of life and preferences in psychosocial support services in this patients group and will also help to identify the characteristics of patients who are most likely to have problems with psychological distress and quality of life. Analyses of data will further provide information about specific problems and supportive care needs of AYA cancer patients in Germany. We will also consider AYA-specific concerns like health behavior, family planning, sexual and reproductive health, significant issues which have received little attention in recent AYA research [8, 25, 26].

As one strengths of the study we are investigating these issues in a broad spectrum of post primary treatment settings (outpatient aftercare, rehabilitation) in a longitudinal design what allows us to keep track of our patients for a longer period than most of the previous longitudinal studies have done [22–24]. Additionally, our expected sample size of *n* = 490 at follow-up will be larger than those to be examined in other studies of AYA cancer patients [19] and will, in contrast to earlier studies, include all malignant cancer types [19]. This fact allows for carrying out subgroup analyses stratified by important risk factors. Finally, our study will provide necessary data from a German sample within the specific German medical care system.

In summary, our longitudinal study will create a sufficient database and will offer detailed information about the life situation and psychosocial care of AYA cancer patients in Germany. Evaluation of existing psychosocial care and patients´ preferences and desires in this area will be used to develop recommendations for the improvement of their psycho-oncological care in the future. Furthermore, our results will provide a basis for creating intervention programs tailored to the unique needs of AYA cancer patients.

## Abbreviations

AYA: Adolescent and young adult
QoL: Quality of life
SPSS: Statistical Package for the Social Sciences
